# Screening for Wilson’s disease in acute liver failure: A new scoring system in children

**DOI:** 10.3389/fped.2022.1003887

**Published:** 2022-09-21

**Authors:** Cai-Xia Feng, Xiu-Qi Chen, Xiao-Li He, Lian-Cheng Lan, Qing Tang, Li Huang, Qing-Wen Shan

**Affiliations:** Department of Pediatrics, The First Affiliated Hospital of Guangxi Medical University, Nanning, China

**Keywords:** acute liver failure, Wilson’s disease, diagnosis, children, scoring system, routine biochemical test

## Abstract

**Background:**

Wilson’s disease (WD) is a rare cause of acute liver failure (ALF) and has a high fatality rate. Rapid and accurate diagnosis is important for ALF because of WD (ALF-WD). Our objective was to establish a simple, rapid, and accurate diagnostic test to distinguish ALF-WD from non-WD ALF (NWDALF) in children.

**Materials and methods:**

The data from all cases with pediatric ALF were retrospectively collected and analyzed. We performed receiver operator characteristics curve (ROC) analysis and confirmed the optimum cut-off points.

**Results:**

Fifty-eight patients with pediatric ALF (12 with WD, 46 with other etiologies) were included. Older age was observed in ALF-WD compared to NWDALF (11.16 ± 2.51 years *vs.* 3.34 ± 3.81 years, *p* < 0.001). An analysis based on routine biochemical testings revealed that total bilirubin (TBil), direct bilirubin, indirect bilirubin, alanine aminotransferase (ALT), aspartate aminotransferase (AST), AST:ALT ratio, alkaline phosphatase (ALP), ALP:TBil ratio, serum albumin, gamma-glutamyl transferase, cholinesterase, hemoglobin, and platelet were statistically significant between the ALF-WD and NWDALF groups. The optimum cut-off points were obtained through ROC analysis. A scoring system was formed by assigning a score of 1 or 0 to patients who met the 13 cut-off points. Using ROC analysis, we determined a cut-off point of ≥ 6.5 for ALF-WD with 91.7% sensitivity and 97.8% specificity (*p* < 0.0001). In addition, a best cut-off point of ≥ 1.5 based on only five variables (ALT, AST, AST:ALT ratio, ALP, and ALP:TBil ratio), had 100% sensitivity and 91.3% specificity for ALF-WD (*p* < 0.0001). Based on this, when age was calculated as the sixth indicator, the best cut-off value of ≥ 2.5 had 100% sensitivity and 97.8% specificity (*p* < 00.0001).

**Conclusion:**

Our study developed a new scoring system that consists of simple laboratory tests with good sensitivity and specificity and can be used by clinicians to quickly distinguish ALF-WD from NWDALF in children.

## Introduction

Wilson’s disease (WD) is an inherited autosomal recessive disorder of copper metabolism that is caused by a pathogenic variant in the ATP7B gene. The clinical manifestations of WD are complex and unpredictable. Acute liver failure (ALF) is a rare but extremely serious clinical type of WD, with a prevalence of 4–36.5% (1–3).

Wilson’s disease is a rare cause of ALF and accounts for about 3% of all ALF (4, 5). In general, without emergency liver transplantation, it comes with high mortality rates (6, 7). Currently, there is a lack of a single rapid diagnostic index for the diagnosis of ALF because of WD (ALF-WD), which makes early diagnosis a challenge. In the ALF setting, the classical Leipzig scoring system (8) has some limitations in the diagnosis of WD. For example, the absence of KF ring in some children with ALF-WD, and the levels of serum ceruloplasmin and 24-h urinary copper may overlap between ALF-WD and non-WD ALF (NWDALF). Furthermore, significant coagulopathy may limit the undertaking of liver biopsy, and ATP7B gene examination takes too long.

Berman et al. (9) first found that an aspartate aminotransferase (AST): alanine aminotransferase (ALT) ratio > 4 and alkaline phosphatase (ALP): total bilirubin (TBil) ratio < 2 had high sensitivity and specificity for the diagnosis of ALF-WD (96–100%). Later, Korman et al. (10) showed that the sensitivity of an AST:ALT ratio > 2.2 and ALP:TBil ratio < 4 to ALF-WD was 94–100% and that the specificity was 86–100%. This suggests that a decrease in the ALP:TBil ratio and increase in the AST:ALT ratio are the laboratory characteristics of ALF-WD and can be used for early and rapid diagnosis. However, the above-mentioned ratio of Korman et al. (10) did not obtain satisfactory results in several subsequent studies of children with ALF-WD, and the specificity was only 19.5–71% (11, 12).

Therefore, further studies are needed to confirm the diagnostic value of these ratios in children. New and accurate diagnostic testing is urgently needed for ALF-WD. In the current study, we present the differences in clinical and biochemical findings between ALF-WD and NWDALF, establishing an accurate and rapid diagnostic test to distinguish ALF-WD from NWDALF.

## Materials and methods

### Patients

**FIGURE 1:**
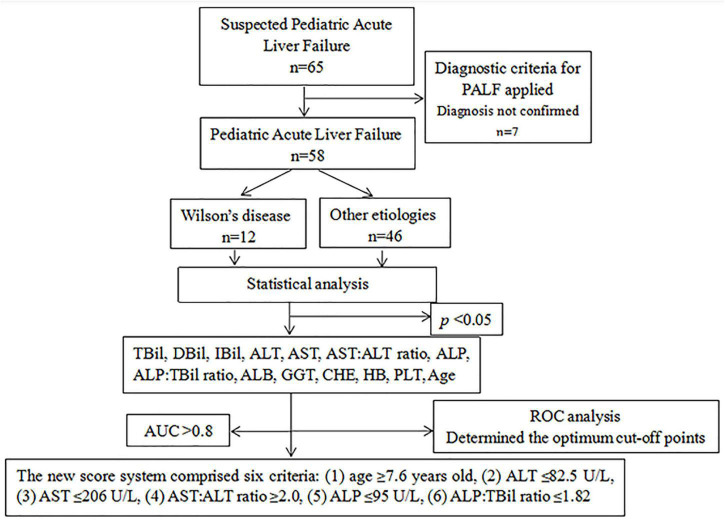
Flow diagram depicting the formation of the new scoring system.

The medical records of 58 patients with pediatric acute liver failure (PALF) who were diagnosed in the First Affiliated Hospital of Guangxi Medical University between December 2012 and January 2022 were retrospectively reviewed (Figure 1).

Inclusion criteria: (1) meet the diagnosis of PALF, (2) children under 18 years old.

Exclusion criteria: (1) patients older than 18 years old, (2) children with serious lack of clinical data.

The diagnostic criteria for PALF (13–16) were as follows: (1) no known history of chronic liver disease, (2) biochemical evidence of acute liver injury, (3) liver-derived coagulopathy and cannot be corrected by vitamin K [defined as prothrombin time (PT) ≥ 15 s or international normalized ratio (INR) ≥ 1.5 in the presence of hepatic encephalopathy or PT ≥ 20 s or INR ≥ 2.0 in the absence of hepatic encephalopathy].

The diagnosis of WD was confirmed according to clinical manifestations, laboratory examination, and was combined with the Leipzig scoring system (8). The Nazer scoring system (17) was calculated for patients with WD.

### Methods

This study set up the case group and the control group. The ALF-WD was classified as the case group, and the NWDALF was classified as the control group. The sex, age, clinical manifestations, outcome and biochemical testings were observed and compared between the two groups.

### Statistical analysis

SPSS version 26.0 was used for the statistical analyses. Continuous variables were defined as the mean ± standard deviation, and categorical variables were defined as frequency or percentages. An unpaired Student’s *t*-test or Wilcoxon rank sum test for continuous variables was used. Pearson Chi-square testing or Fisher’s exact values for categorical variables were used. After identifying the significant differences between the two groups, a receiver operator characteristics curve (ROC) analysis was performed for the variables with *p* < 0.05. The sensitivity, specificity, and likelihood ratio were calculated for each variable, and the optimum cut-off points were determined. A scoring system was formed by assigning a score of 1 or 0 to patients who met the these cut-off points. Finally, we chose all the areas under the ROC curve higher than 0.80 for further examination, and a new scoring system was identified based on these variables.

## Results

### Clinical features of the patient groups

Wilson’s Disease patient cohort: Among the 12 patients with ALF-WD, 10 patients had ceruloplasmin < 200 mg/L and Coombs-negative hemolytic anemia. Seven patients had 24-h urinary copper levels > 100 μg. K-F rings were present in nine patients. Five patients presented with one or more ATP7B variants (Table 1).

**TABLE 1:** Clinical and biochemical characteristics of ALF because of WD in 12 cases.

Case	Sex	Age (year)	Hemolytic anemia	K-F ring	Cp	U-Copper	Mutation	Nazer score	Outcome
1	F	9.0	Yes	Pos	198.7	ND	c.2111C > T, p.T704I	7	Death
2	F	10.8	Yes	Pos	172.1	869.3	c.747G > A, p.L249L c.3443T > C, p.I1148T	9	Death
3	F	10.3	Yes	Pos	107	24.1	c.1168A > G, p.I390V) c.1708–1G > C	7	Alive
4	F	14.5	Yes	Pos	182	4883.2	c.4112T > C, p.L1371P)	6	Alive
5	F	11.8	Yes	Pos	120	2091.7	ND	10	Death
6	M	12.9	No	Pos	124	986.2	c.1040delG, p.G347fs c.2828G > A, p.G943D	3	Alive
7	M	9.2	No	Pos	76	41.9	ND	7	Alive
8	F	14.7	Yes	Pos	55.8	19.7	ND	9	Death
9	M	9.1	Yes	Pos	165	172.1	ND	7	Death
10	F	14.8	Yes	ND	288	641.4	ND	10	Death
11	M	8.7	Yes	ND	50.5	ND	ND	12	Death
12	F	8.2	Yes	Neg	247.9	518.2	ND	7	Death

ALF, acute liver failure; WD, Wilson’s disease; K-F, Kayser–Fleischer; Cp, ceruloplasmin (mg/L); U-Copper, 24-h urinary copper(ug); F, female; M, male; Pos, positive; Neg, negative; ND, not done.

Other disease groups: The 46 patients with NWDALF included unknown etiology (*n* = 26; 56.5%), hereditary metabolic diseases (*n* = 7; 15.2%), infectious causes (*n* = 3; 6.5%), autoimmune hepatitis (*n* = 3; 6.5%), hemophagocytic syndrome (*n* = 2; 4.3%), liver ischemia-induced (*n* = 2; 4.3%), langerhans cell histiocytosis (*n* = 1; 2.2%), liver failure because of neoplasm and drug-induced (*n* = 1; 2.2%).

Among the 12 patients with ALF-WD, the mean age was 11.16 ± 2.51 years (range: 8.2–14.7), and eight were female. Among the 46 patients with NWDALF, the mean age was 3.34 ± 3.81 years (range: 0.04–12.7), including 17 female. Older age (*p* < 0.001) and higher incidence of splenomegaly, ascites, and liver cirrhosis (*p* = 0.02, *p* = 0.042, and *p* = 0.001) were observed in patients with ALF-WD (Table 2).

**TABLE 2:** Comparison of clinical manifestations between ALF-WD and NWDALF.

Clinical manifestation	ALF-WD (*n* = 12)	NWDALF (*n* = 46)	*P*-value
Gender [*n* (%)]			
Male	4 (33.3%)	29 (63%)	0.064
Female	8 (66.7%)	17 (37%)	
Age (year)	11.16 ± 2.51	3.34 ± 3.81	< 0.001
Hepatomegaly [*n* (%)]	5 (41.7%)	27 (58.7%)	0.291
Splenomegaly [*n* (%)]	9 (75%)	15 (32.6%)	0.020
Ascites [*n* (%)]	9 (75%)	17 (37%)	0.042
Liver cirrhosis [*n* (%)]	7 (58.3%)	5 (10.9%)	0.001
Hepatic encephalopathy [*n* (%)]	6 (50%)	23 (50%)	1
Gastrointestinal hemorrhage [*n* (%)]	4 (33.3%)	10 (21.7%)	0.648
Pulmonary hemorrhage [*n* (%)]	5 (41.7%)	9 (19.6%)	0.225
Multiple organ failure [*n* (%)]	6 (50%)	24 (52.2%)	0.893

ALF-WD, acute liver failure because of Wilson’s disease; NWDALF, non-WD acute liver failure.

### Copper metabolism parameters

Compared with patients with NWDALF, patients with ALF-WD had a lower serum ceruloplasmin and a higher 24-h urinary copper (*p* = 0.002 and *p* = 0.037, respectively). There was no significant difference in the serum copper between ALF-WD and NWDALF (*p* = 0.412) (Table 3). A ROC analysis of ceruloplasmin found that the best cut-off point < 199.6 mg/L for ALF-WD had an area under the curve (AUC) of 0.8241 (*p* = 0.003), a sensitivity of 88.3%, a specificity of 77.8%, a 3.75 positive likelihood ratio, and a 0.21 negative likelihood ratio. A ROC analysis of 24-h urinary copper showed that a best cut-off point > 166.1 μg for ALF-WD had an AUC of 0.7938 (*p* = 0.0368), a sensitivity of 70%, a specificity of 100%, and a 0.3 negative likelihood ratio.

**TABLE 3:** Comparison of copper metabolism parameters of children with ALF-WD or NWDALF.

Variable	ALF-WD (*n* = 12)	NWDALF (*n* = 46)	*P*-value
Serum ceruloplasmin (mg/L)	148.92 ± 70.1	248.34 ± 78.85	0.002
24-h urinary copper (μg/24 h)	1024.76 ± 1496.64	61.71 ± 56.9	0.037
Serum copper (μmol/L)	15.95 ± 9.64	11.52 ± 4.61	0.412

ALF-WD, acute liver failure because of Wilson’s disease; NWDALF, non-WD acute liver failure.

### Laboratory tests

An analysis based on routine biochemical testing revealed that the serum levels of TBil, direct bilirubin (DBil), indirect bilirubin (IBil), AST:ALT ratio, and gammaglutamyl transferase (GGT) were significantly higher in ALF-WD than in NWDALF (*p* = 0.018, *p* = 0.021, *p* = 0.044, *p* < 0.001, and *p* = 0.032, respectively). ALT, AST, serum albumin (ALB), ALP, ALP:TBil ratio, cholinesterase (CHE), hemoglobin (Hb), and platelet (PLT) levels were significantly lower in the ALF-WD group compared with NWDALF group (*p* < 0.001, *p* < 0.001, *p* = 0.013, *p* < 0.001, *p* < 0.001, *p* = 0.014, *p* = 0.026, and *p* = 0.015, respectively) (Table 4).

**TABLE 4:** Comparison of laboratory tests of children with ALF-WD or NWDALF.

Variable	ALF-WD (*n* = 12)	NWDALF (*n* = 46)	*P*-value
TBil (μmol/L)	516.57 ± 319.03	294.62 ± 206.73	0.018
Direct bilirubin (μmol/L)	297.43 ± 193.53	162.63 ± 120.14	0.021
Indirect bilirubin (μmol/L)	219.14 ± 141.02	131.98 ± 101.37	0.044
ALT (U/L)	41.75 ± 21.77	1087.54 ± 2212.26	< 0.001
AST (U/L)	171.42 ± 97.13	1413.15 ± 3094.70	< 0.001
AST:ALT ratio	4.67 ± 2.36	1.96 ± 1.88	< 0.001
Serum albumin (g/L)	28.45 ± 4.64	33.95 ± 7.04	0.013
ALP (U/L)	92.92 ± 134.26	407.11 ± 267.60	< 0.001
ALP:TBil ratio	15.33 ± 36.15	55.20 ± 81.62	< 0.001
Gammaglutamyl transferase (U/L)	144.33 ± 96.53	110.93 ± 175.28	0.032
Cholinesterase (U/L)	3259.00 ± 2185.37	5029.70 ± 2381.09	0.014
Prothrombin time(s)	31.89 ± 11.26	36.75 ± 25.19	0.992
International normalized ratio	2.69 ± 0.99	3.00 ± 2.04	0.853
Activated partial prothrombin time(s)	60.11 ± 30.70	61.44 ± 23.27	0.496
Fibrin (g/L)	1.37 ± 0.36	1.44 ± 0.67	0.985
Prothrombin activity (%)	27.00 ± 9.00	29.66 ± 15.87	0.827
White blood cells (10^9^/L)	12.78 ± 7.43	13.7 ± 8.89	0.742
Hemoglobin (g/L)	77.14 ± 24.90	94.95 ± 23.87	0.026
Platelet (10^9^/L)	116.09 ± 57.19	206.51 ± 132.43	0.015
Blood ammonia (μmol/L)	143.50 ± 83.67	195.83 ± 121.36	0.17
Blood lactic acid (mmol/L)	3.14 ± 1.53	5.15 ± 5.11	0.107

ALF-WD, acute liver failure because of Wilson’s disease; NWDALF, non-WD acute liver failure; TBil, total bilirubin; ALT, alanine aminotransferase; AST, aspartate aminotransferase; ALP, alkaline phosphatase.

### Screening for Wilson disease in acute liver failure

The optimum cut-off points for the above 13 routine biochemical testing in ALF-WD were obtained with a ROC analysis (Table 5). A scoring system was formed by assigning a score of 1 or 0 to patients who met these cut-off points. Using ROC analysis, we obtained a best cut-off point of ≥ 6.5 for ALF-WD. The AUC was 0.9728, with 91.7% sensitivity, 97.8% specificity, 42.17 positive likelihood ratio, and 0.09 negative likelihood ratio (*p* < 0.0001).

**TABLE 5:** Determination of the optimal cut-off points of laboratory tests for ALF-WD.

Variable	AUC	Cut-off value	Sensitivity	Specificity	*LR*+	*LR−*	*P*-value for AUC
TBil	0.7228	≥ 420.5 μmol/L	66.7%	82.6%	3.83	0.40	0.0182
DBi	0.7174	≥ 288.2 μmol/L	50%	93.5%	7.67	0.53	0.0213
IBi	0.6902	≥ 267.1 μmol/L	50%	91.3%	5.75	0.55	0.0439
ALT	0.9683	≤ 82.5 U/L	100%	91.3%	11.5	0.00	< 0.0001
AST	0.8659	≤ 206 U/L	75%	84.8%	4.93	0.29	0.0001
AST:ALT Ratio	0.8514	≥ 2.0	91.7%	69.6%	3.01	0.12	0.0002
ALB	0.7446	≤ 30.75 g/L	75%	67.4%	2.30	0.37	0.0096
ALP	0.8967	≤ 95 U/L	75%	100%	NC	0.25	< 0.0001
ALP:TBil Ratio	0.8478	≤ 1.82	66.7%	100%	NC	0.33	0.0002
GGT	0.7029	≥ 110.5 U/L	75%	73.9%	2.88	0.34	0.0316
CHE	0.7319	≤ 3109 U/L	66.7%	80.4%	3.41	0.41	0.014
Hb	0.7183	≤ 77 g/L	66.7%	78.3%	3.07	0.43	0.0207
PLT	0.7292	≤ 122 × 10^9^/L	75%	69.6%	2.46	0.36	0.0152

ALF-WD, acute liver failure because of Wilson’s disease; NWDALF, non-WD acute liver failure; TBil, total bilirubin; DBil, direct bilirubin; IBil, indirect bilirubin; ALT, alanine aminotransferase; AST, aspartate aminotransferase; ALB, albumin; ALP, alkaline phosphatase; GGT, gammaglutamyl transferase; CHE, cholinesterase; Hb, hemoglobin; PLT, platelet; LR+, positive likelihood ratio; LR−, negative likelihood ratio; NC, not calculate.

A combination of five variables with the largest AUC, including ALT, AST, ALP, AST:ALT ratio, and ALP:TBil ratio, had the best cut-off point of ≥ 1.5 for ALF-WD, which had 100% sensitivity, 91.3% specificity, 11.5 positive likelihood ratio, 0.00 negative likelihood ratio, and an AUC of 0.9937 (*p* < 0.0001). There was a significant difference in age between ALF-WD and NWDALF, so we conducted a ROC analysis of age. Then, a cut-off point of ≥ 7.6 was determined with 100% sensitivity, 84.8% specificity, 6.57 positive likelihood ratio, 0.00 negative likelihood ratio, and an AUC of 0.9248 (*p* < 0.0001). Based on this, when age was calculated as the sixth indicator, a best cut-off value of ≥ 2.5 had 100% sensitivity, 97.8% specificity, 46 positive likelihood ratio, 0.00 negative likelihood ratio, and AUC of 0.9964 (*p* < 0.0001). This was the combination of the highest diagnostic values for ALF-WD. Therefore, age, ALT, AST, AST:ALT ratio, ALP, and ALP:TBil ratio were used as the new scoring system for the diagnosis of ALF-WD (Table 6).

**TABLE 6:** Scoring system for the diagnosis of ALF-WD.

Variable	Cut-off value for ALF-WD	Score
Age (year)	≥ 7.6	1
	< 7.6	0
ALT (U/L)	≤ 82.5	1
	> 82.5	0
AST (U/L)	≤ 206	1
	> 206	0
AST:ALT ratio	≥ 2.0	1
	< 2.0	0
ALP (U/L)	≤ 95	1
	> 95	0
ALP:TBil ratio	≤ 1.82	1
	> 1.82	0

A combination of five variables (ALT, AST, AST:ALT ratio, ALP, and ALP:TBil ratio) had a best cut-off point of ≥ 1.5, which had 100% sensitivity, 91.3% specificity, and an AUC of 0.9937 (*p* < 0.0001). When age was calculated as the sixth indicator, a best cut-off value of ≥ 2.5 had 100% sensitivity, 97.8% specificity, and an AUC of 0.9964 (*p* < 00.0001). ALT, alanine aminotransferase; AST, aspartate aminotransferase; ALP, alkaline phosphatase; TBil, total bilirubin.

### Validation of our new scoring system

A review of the Chinese literature and English literature was performed to determine whether our new scoring system applied to other cases with ALF-WD. Some cases reported in the literature could not be included because the patients were older than 18 years old and some necessary biochemical parameters used in the scoring system were absent. Finally, a total of 36 children with ALF-WD were enrolled in the validation cohort, including 5 children in our center. In the validation cohort, we can see that 100% of the patients had a score of ≥ 2.5 (Supplementary Table 1). This was suggested that our scoring system also has high accuracy in other cases with ALF-WD.

### Comparison with the existing scoring system

**FIGURE 2:**
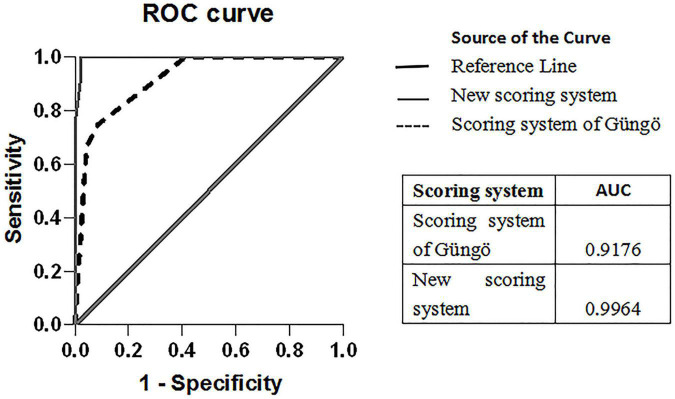
ROC curves of the scoring system of Güngö et al. and our scoring system for ALF-WD. The comparison of the area under ROC for the scoring system is depicted in the box above.

The scoring system of Güngö et al. (11) comprised hemoglobin, platelet, albumin, cholesterol, low density lipoprotein, uric acid, TBil, DBil, GGT, ALP, ALT, AST, AST:ALT ratio, and ALP:TBil ratio. When the score was ≥ 4.5, it had 100% sensitivity in our study population, but the specificity was only 58.7%. A scoring system composed of hemoglobin, uric acid, ALP, ALT, AST, and AST:ALT ratio was used. When the score was ≥ 2.5, the sensitivity was 100% in our study population, but the specificity was only 58.7%. The ROC analysis of Güngö et al. (11) and our scoring system is shown in Figure 2.

### Outcomes

Four of the 12 patients with ALF-WD were treated with plasma exchange (PE) and chelation therapy and survived without liver transplantation. Using the Nazer scoring system (17), two of the four patients scoring < 7 survived. The remaining eight patients who died without liver transplantation had a score of ≥ 7 (Table 1).

## Discussion

It is a challenge to rapidly diagnose ALF-WD because of the lack of sensitive and specific diagnostic criteria. However, early diagnosis of ALF-WD is vitally important because of its high mortality and poor prognosis (18). In general, a decrease of ceruloplasmin, increase of 24-h urine copper, elevated hepatic copper, and presence of K-F ring are regarded as strong indications of WD. However, a decrease of ceruloplasmin was not found to be specific to patients with WD, and it could also be observed in some patients with NWDALF (10, 19). In addition, in the ALF setting, there may be a high overlap in urinary copper between ALF-WD and NWDALF. Furthermore, significant coagulopathy may limit the undertaking of liver biopsy. In addition, some patients with ALF-WD are absence of KF ring. A meta-analysis of 256 children with ALF-WD showed that K-F rings were present in about 74% of these children (20). In addition, some patients are unable to undergo slit lamp examinations because of their critical condition. The above-mentioned limitations of these classical tests make it difficult to diagnose ALF-WD. Thus, the diagnostic utility of these classical diagnostic tests needed to be re-evaluated.

Serum ceruloplasmin is a copper carrier protein that binds to 90% of circulating copper in normal people. The level of ceruloplasmin in patients with WD has been found to decrease significantly, which was first confirmed by Scheinberg and Gitlin (21) in 1952. In general, the level of ceruloplasmin in most children with WD is less than 20 mg/dL (22, 23). In children with ALF-WD, a meta-analysis that included 256 cases showed that the average level of serum ceruloplasmin was 11 mg/dL (20). However, in both WD and other causes of ALF, ceruloplasmin greatly fluctuates, and it can be normal or elevated. Many studies have shown that there was no significant difference in ceruloplasmin between ALF-WD and NWDALF (10, 19, 24). On the contrary, Güngö et al. (11) suggested that children with ALF-WD had lower ceruloplasmin compared with children with NWDALF. At the same time, a cut-off value of < 19.5 mg/dL of ceruloplasmin with 81.4% sensitivity and 100% specificity in distinguishing ALF-WD and NWDALF was suggested. Our study showed that children with ALF-WD have lower ceruloplasmin. A best cut-off value of < 199.6 mg/L was used for ALF-WD, with a sensitivity of 83.3% and specificity of 77.8%. Compared with the study by Güngö et al. (11), a similar cut-off value of ceruloplasmin was observed, but the specificity was lower in our study. This indicates that the diagnostic value of ceruloplasmin in children is still controversial and needs further verification.

Increased urinary copper excretion is an important feature of WD, and some studies have shown a significant increase in 24-h urinary copper levels in children with ALF-WD (12, 20, 25). However, it is worth noting that 24-h urinary copper also increases significantly in other related liver diseases that are characterized by a large number of hepatocyte necrosis, so there may be a large overlap between ALF-WD and ALF with other etiology. A study including 24 children with ALF-WD and 120 children with NWDALF showed that 24-h urine copper ≥ 239.5 μg in children with ALF-WD had a sensitivity of 91.7% and specificity of 100% (11). However, those results have not yet been further verified and applied. Our study revealed that children with ALF-WD had higher 24-h urine copper levels than children with NWDALF. A best cut-off value of ≥ 166.1 μg was used for diagnosing ALF-WD, with 70% sensitivity and 100% specificity. We can attribute our lower 24-h urine copper cut-off value than those reported before to the fact that 7 of the 12 patients with WD had the complication of acute renal failure. In acute renal failure, the decrease in urine volume may affect the collection of urine and determination of urinary copper.

Because of the limitations of classical diagnostic tests in the ALF setting, routine biochemical indicators for the diagnosis of ALF-WD have received increasing attention. Previous studies have attempted to use simple biochemical markers to diagnose ALF-WD and have obtained new findings. Berman et al. (9) first found that an ALP:TBil ratio < 2 and AST:ALT ratio > 4 were of great value in the diagnosis of ALF-WD. The ALP:TBil ratio < 2 had 100% sensitivity and specificity and AST:ALT ratio > 4 had 83.3% sensitivity and 100% specificity, respectively. Korman et al. (10) showed that the sensitivity and specificity of ALF-WD diagnosed by an ALP:TBil ratio < 4 and AST:ALT ratio > 2.2 were 94–98% and 86–100%, respectively. The above-mentioned ratios were calculated for adults, which means that the diagnostic value has not been validated in children. In our study, we noted that the sensitivity and specificity of these two ratios in children were lower than those in adults, and the reason for this cannot be explained currently. Similar findings have been found in ALF-WD studies of children, which revealed that the specificity was only 19.5–71% (11, 12).

Coombs-negative hemolytic anemia is another feature of ALF caused by WD, and it can be observed in children with ALF-WD (11, 12, 20, 26). In the classic case of ALF-WD, the sudden release of copper from the liver leads to high levels of non-ceruloplasmin-bound copper in plasma, resulting in excessive destruction of red blood cells, which further leads to intravascular hemolysis. In the present study, hemolytic anemia was observed in 10 of 12 children with ALF-WD. The hemoglobin levels of children with ALF-WD were lower than NWDALF, and the difference was statistically significant. However, hemoglobin as an index to distinguish ALF-WD from NWDALF had low sensitivity and specificity. Our study showed that a cut-off point of < 77 g/L for hemoglobin confirmed the diagnosis of ALF-WD with 66.7% sensitivity and 78.3% specificity. This is similar to the study of Güngö et al. (11), which found that a cut-off point of < 91.5 g/L for hemoglobin had 66.7% sensitivity and 76.7% specificity. Because many factors affect the level of hemoglobin, there are some limitations in the diagnosis of ALF-WD. In the future, a comprehensive study on Coombs-negative hemolytic anemia in ALF-WD is needed.

Individual routine biochemical markers are limited in diagnosing ALF-WD. Güngö et al. (11) suggested that combining the results of multiple laboratory tests can help to quickly diagnose ALF-WD in children. When the variable values calculated by 14 indexes (hemoglobin, platelet, albumin, cholesterol, low density lipoprotein, uric acid, TBil, DBil, GGT, ALT, AST, ALP, AST:ALT ratio, and ALP:TBil ratio) were ≥ 4.5, the sensitivity was 88.9% and specificity 87.9%. When the variable values calculated by six indexes (hemoglobin, uric acid, ALT, AST, ALP, and AST/ALT) were ≥ 2.5, the sensitivity was 87.5% and specificity 86.7%. However, the above method has not been further verified. In our study, we found that this scoring system did not obtain satisfactory results, with the specificity only being 58.7%. Compared with the scoring system of Güngö et al. (11), our new scoring system had higher sensitivity and specificity.

It has been pointed out that there will be a high mortality rate of ALF-WD if liver transplantation is not performed (6, 7). However, some studies have found that the treatment of non–liver transplantation, including blood purification and chelation therapy, can also survive children with ALF-WD (12, 27–30). In our study, 4 of 12 patients with ALF-WD survived without liver transplantation. Nazer et al. (17) developed a scoring system to evaluate the prognosis of patients who did not undergo liver transplantation. When the score was used in our study, only two patients did not meet expectations. These two patients with a score of 7 and who were predicted to die instead survived. Compared with liver transplantation, blood purification and chelation therapy are easy to obtain, which are early strategies for the treatment of ALF-WD and which can save some children’s lives, but more studies are needed to further verify this. According to the current research, liver transplantation is still the most important treatment method to save children’s lives and improve their long-term survival rate (31–33).

The strength of the new scoring system is that it consists of simple laboratory tests with good sensitivity and specificity and can be used by clinicians to quickly distinguish ALF-WD from NWDALF. Rapid diagnosis is important for children who can benefit from chelating treatment. One limitation of our study is that, because of the rarity of WD presenting as ALF, a relatively small number of children with ALF-WD were enrolled.

In conclusion, ALF-WD in children is a rare clinical manifestation of WD, with rapid progress and a high fatality rate, so early diagnosis is crucial. Currently, there is no single rapid diagnostic index for ALF-WD. The importance of routine biochemical tests in the diagnosis of ALF-WD has been attracting increasing attention. Our study established a new scoring system with 100% sensitivity and 97.8% specificity in children to make a diagnosis of ALF-WD.

## Data availability statement

The original contributions presented in this study are included in the article/Supplementary material, further inquiries can be directed to the corresponding author.

## Ethics statement

Written informed consent was obtained from the individual(s) for the publication of any potentially identifiable images or data included in this article.

## Author contributions

C-XF and Q-WS conceived and designed the study. C-XF, X-QC, X-LH, L-CL, QT, and LH collected the clinical data of the patients and drafted manuscript. Q-WS approved the final manuscript. All authors contributed to the article and approved the submitted version.

## References

[1] Rukunuzzaman M. Wilson’s disease in bangladeshi children: analysis of 100 cases. *Pediatr Gastroenterol Hepatol Nutr.* (2015) 18:121. 10.5223/pghn.2015.18.2.121 26157698 PMC4493245

[2] Dhawan A Taylor RM Cheeseman P De Silva P Katsiyiannakis L Mieli-Vergani G. Wilson’s disease in children: 37-year experience and revised King’s score for liver transplantation. *Liver Trans.* (2005) 11:441–8. 10.1002/lt.20352 15776453

[3] Sintusek P Chongsrisawat V Poovorawan Y. Wilson’s disease in thai children between 2000 and 2012 at king chulalongkorn memorial hospital. *J Med Assoc Thai.* (2016) 99:182–7. 27249898

[4] Squires RH Shneider BL Bucuvalas J Alonso E Sokol RJ Narkewicz MR Acute liver failure in children: the first 348 patients in the pediatric acute liver failure study group. *J Pediatr.* (2006) 148:652–658e2. 10.1016/j.jpeds.2005.12.051 16737880 PMC2662127

[5] Ostapowicz G Fontana RJ Schiødt FV Larson A Davern TJ Han SHB Results of a prospective study of acute liver failure at 17 tertiary care centers in the United States. *Ann Intern Med.* (2002) 137:947. 10.7326/0003-4819-137-12-200212170-00007 12484709

[6] Mainardi V Rando K Valverde M Olivari D Castelli J Rey G Acute liver failure due to wilson disease: eight years of the national liver transplant program in uruguay. *Ann Hepatol.* (2019) 18:187–92. 10.5604/01.3001.0012.7911 31113589

[7] Mendizabal M Dip M Demirdjian E Lauferman L Lopez S Minetto J Changing etiologies and prognostic factors in pediatric acute liver failure. *Liver Trans.* (2020) 26:268–75. 10.1002/lt.25658 31606931

[8] Ferenci P Caca K Loudianos G Mieli-Vergani G Tanner S Sternlieb I Diagnosis and phenotypic classification of Wilson disease. *Liver Int.* (2003) 23:139–42. 12955875 10.1034/j.1600-0676.2003.00824.x

[9] Berman DH Leventhal RI Gavaler JS Cadoff EM Van Thiel DH. Clinical differentiation of fulminant Wilsonian hepatitis from other causes of hepatic failure. *Gastroenterology.* (1991) 100:1129. 10.1016/0016-5085(91)90294-u 2001814

[10] Korman JD Volenberg I Balko J Webster J Schiodt FV Squires RH Screening for wilson disease in acute liver failure: a comparison of currently available diagnostic tests. *Hepatology.* (2008) 48:1167–74. 10.1002/hep.22446 18798336 PMC4881751

[11] Gungor S Selimoglu MA Bag H Varol FI. Is it possible to diagnose fulminant Wilson’s disease with simple laboratory tests? *Liver Int.* (2020) 40:155–62. 10.1111/liv.14263 31568639

[12] Fang W Abuduxikuer K Shi P Qiu Y Zhao J Li Y Pediatric Wilson disease presenting as acute liver failure: prognostic indices. *World J Clin Cases.* (2021) 9:3273–86. 10.12998/wjcc.v9.i14.3273 34002136 PMC8107887

[13] Dhawan A. Acute liver failure in children and adolescents. *Clin Res Hepatol Gastroenterol.* (2012) 36:278–83. 10.1016/j.clinre.2012.03.022 22521555

[14] Squires JE McKiernan P Squires RH. Acute liver failure: an update. *Clin Liver Dis.* (2018) 22:773–805. 10.1016/j.cld.2018.06.009 30266162

[15] Kathemann S Bechmann LP Sowa JP Manka P Dechêne A Gerner P Etiology, outcome and prognostic factors of childhood acute liver failure in a German single center. *Ann Hepatol.* (2015) 14:722–8. 26256901

[16] Squires JE Alonso EM Ibrahim SH Kasper V Kehar M Martinez M North American society for pediatric gastroenterology, hepatology, and nutrition position paper on the diagnosis and management of pediatric acute liver failure. *J Pediatr Gastroenterol Nutr.* (2022) 74:138–58. 10.1097/MPG.0000000000003268 34347674

[17] Nazer H Ede RJ Mowat AP Williams R. Wilson’s disease: clinical presentation and use of prognostic index. *Gut.* (1986) 27:1377–81. 10.1136/gut.27.11.1377 3792921 PMC1434058

[18] Patil M Sheth KA Krishnamurthy AC Devarbhavi H. A review and current perspective on Wilson disease. *J Clin Exp Hepatol.* (2013) 3:321–36. 10.1016/j.jceh.2013.06.002 25755520 PMC3940372

[19] Eisenbach C Sieg O Stremmel W Encke J Merle U. Diagnostic criteria for acute liver failure due to Wilson disease. *World J Gastroenterol.* (2007) 13:1711–4. 10.3748/wjg.v13.i11.1711 17461475 PMC4146951

[20] Vandriel SM Ayoub MD Ricciuto A Hansen BE Ling SC Ng VL Pediatric Wilson disease presenting as acute liver failure: an individual patient data meta-analysis. *J Pediatr Gastrenterol Nutr.* (2020) 71:e90–6. 10.1097/MPG.0000000000002777 32520831

[21] Scheinberg IH Gitlin D. Deficiency of ceruloplasmin in patients with hepatolenticular degeneration (Wilson’s disease). *Science.* (1952) 116:484–5. 10.1126/science.116.3018.484 12994898

[22] Li X Lu Z Lin Y Lu X Xu Y Cheng J Clinical features and mutational analysis in 114 young children with Wilson disease from South China. *Am J Med Genet.* (2019) 179:1451–8. 10.1002/ajmg.a.61254 31172689

[23] Nicastro E Ranucci G Vajro P Vegnente A Iorio R. Re-evaluation of the diagnostic criteria for Wilson disease in children with mild liver disease. *Hepatology.* (2010) 52:1948–56. 10.1002/hep.23910 20967755

[24] Mak CM Lam C Tam S. Diagnostic accuracy of serum ceruloplasmin in Wilson disease: determination of sensitivity and specificity by ROC curve analysis among ATP7B-genotyped subjects. *Clin Chem.* (2008) 54:1356–62. 10.1373/clinchem.2008.103432 18556333

[25] Devarbhavi H Singh R Adarsh CK Sheth K Kiran R Patil M. Factors that predict mortality in children with Wilson disease associated acute liver failure and comparison of Wilson disease specific prognostic indices. *J Gastroenterol Hepatol.* (2014) 29:380–6. 10.1111/jgh.12356 24033813

[26] Pop TL Grama A Stefanescu AC Willheim C Ferenci P. Acute liver failure with hemolytic anemia in children with Wilson’s disease: genotype-phenotype correlations? *World J Hepatol.* (2021) 13:1428–38. 10.4254/wjh.v13.i10.1428 34786177 PMC8568583

[27] Verma N Pai G Hari P Lodha R. Plasma exchange for hemolytic crisis and acute liver failure in Wilson disease. *Indina J Pediatr.* (2013) 81:498–500. 10.1007/s12098-013-0979-x 23494839

[28] Zhang Y Li L Zhang X Xu W Guo Q Zhou J. Plasmapheresis combined with continuous plasma filtration adsorption rescues severe acute liver failure in Wilson’s disease before liver transplantation. *Blood Purif.* (2018) 47:120–5. 10.1159/000493909 30359967

[29] Damsgaard J Larsen FS Ytting H. Reversal of acute liver failure due to wilson disease by a regimen of high-volume plasma exchange and penicillamine. *Hepatology.* (2019) 69:1835–7. 10.1002/hep.30323 30357869

[30] Pawaria A Sood V Lal BB Khanna R Bajpai M Alam S. Ninety days transplant free survival with high volume plasma exchange in Wilson disease presenting as acute liver failure. *J Clin Apher.* (2021) 36:109–17. 10.1002/jca.21848 33368534

[31] Arnon R Annunziato R Schilsky M Miloh T Willis A Sturdevant M Liver transplantation for children with Wilson disease: comparison of outcomes between children and adults. *Clin Transplant.* (2011) 25:E52–60. 10.1111/j.1399-0012.2010.01327.x 20946468

[32] Guillaud O Dumortier J Sobesky R Debray D Wolf P Vanlemmens C Long term results of liver transplantation for Wilson’s disease: experience in France. *J Hepatol.* (2014) 60:579–89. 10.1016/j.jhep.2013.10.025 24211743

[33] Ismail MS Hassan M Martinez-Camacho A May SB Goss JA Kanwal F Retrospective analysis of long-term outcome 10 years after liver transplantation for Wilson disease: experience over three decades. *Transpl Int.* (2020) 33:925–35. 10.1111/tri.13622 32314442

