# Supplementary material for: Screening for Wilson’s disease in acute liver failure: A new scoring system in children
Source: Front Pediatr. 2022 Sep 21;10:1003887. doi: 10.3389/fped.2022.1003887 (PMC9534029; doi:10.3389/fped.2022.1003887)
Supplement: Supplementary file 1 [file Table_1.DOCX]

Supplementary Table 1. Cases of ALF-WD in the validation cohort

| Case | Sex | ALT (U/L) | AST (U/L) | AST:ALT ratio | ALP (U/L) | TBil  (mg/dl) | ALP:TBil ratio | Score |
| --- | --- | --- | --- | --- | --- | --- | --- | --- |
| 1 | Female | 11 | 165 | 15.0 | 18 | 53.4 | 0.3 | 5 |
| 2 | Female | 29 | 250 | 8.6 | 28 | 40.0 | 0.7 | 5 |
| 3 | Female | 44 | 83 | 1.9 | 43 | 5.2 | 8.3 | 4 |
| 4 | Female | 15 | 123 | 8.2 | 11 | 38.7 | 0.3 | 6 |
| 5 | Female | 29 | 162 | 5.6 | 30 | 44.0 | 0.7 | 6 |
| 6 | Male | 110 | 230 | 2.1 | 15 | 41.0 | 0.4 | 4 |
| 7 | Female | 235 | 176 | 0.7 | 4.2 | 45.0 | 0.0 | 4 |
| 8 | Female | 15 | 118 | 7.9 | 32 | 55.0 | 0.6 | 6 |
| 9 | Female | 28 | 85 | 3.0 | 58 | 52.0 | 1.1 | 6 |
| 10 | Male | 89 | 109 | 1.2 | 92 | 38.0 | 2.4 | 3 |
| 11 | Female | 131 | 345 | 2.6 | 103 | 58.0 | 1.8 | 3 |
| 12 | Female | 63 | 44 | 0.7 | 195 | 66.0 | 2.9 | 3 |
| 13 | Male | 23 | 251 | 10.9 | NA | NA | 0.6 | 4 |
| 14 | Female | 64 | 142 | 2.2 | NA | NA | 3.7 | 4 |
| 15 | Male | 10 | 124 | 12.4 | NA | NA | 0.3 | 5 |
| 16 | Female | 18 | 194 | 10.8 | NA | NA | 0.4 | 5 |
| 17 | Female | 14 | 74 | 5.3 | 31 | 31.1 | 1.0 | 5 |
| 18 | Male | 28.8 | 95.5 | 3.3 | 46.1 | 51.7 | 0.9 | 5 |
| 19 | Male | 16.7 | 234.2 | 14.0 | 19.8 | 35.7 | 0.6 | 5 |
| 20 | Female | 13.8 | 124 | 9.0 | 6.3 | 50.6 | 0.1 | 6 |
| 21 | Female | 55 | 112.5 | 2.0 | 54.8 | 31.2 | 1.8 | 6 |
| 22 | Female | 42 | 161 | 3.8 | 2.0 | 48.5 | 0.0 | 6 |
| 23 | Male | 22.4 | 154.9 | 6.9 | 19.4 | 35.7 | 0.5 | 6 |
| 24 | Female | 21.2 | 114.5 | 5.4 | 13.2 | 30.5 | 0.4 | 6 |
| 25 | Female | 20 | 114 | 5.7 | 15 | 38.9 | 0.4 | 5 |
| 26 | Female | 19 | 131 | 6.9 | 662 | 28.0 | 23.6 | 4 |
| 27 | Male | 32 | 153 | 4.8 | 42 | 31.7 | 1.3 | 6 |
| 28 | Female | 55 | 138 | 2.5 | 108 | 34.8 | 3.1 | 4 |
| 29 | Female | 11 | 136 | 12.4 | 9 | 40.3 | 0.2 | 6 |
| 30 | Male | 22.9 | NA | NA | 9.3 | 32.1 | 0.3 | 4 |
| 31 | Male | 67 | 330 | 4.9 | 71 | 24.0 | 3.0 | 4 |
| 32 | Male | 66 | 101 | 0.7 | 274 | 47.0 | 5.8 | 3 |
| 33 | Female | 18 | 50 | 2.8 | 136 | 11.3 | 12.0 | 4 |
| 34 | Female | 28 | 48 | 1.7 | 121 | 4.7 | 25.9 | 3 |
| 35 | Male | 135 | 119 | 0.9 | 80 | 2.4 | 32.8 | 3 |
| 36 | Female | 29 | 68 | 2.3 | 51 | 22.0 | 2.3 | 5 |

*ALF-WD,* acute liver failure because of Wilson’s disease; *ALT,* alanine aminotransferase; *AST,* aspartate aminotransferase; *ALP,* alkaline phosphatase; *TBil,* total bilirubin; *NA*, not applicable.
